# Twenty Five–Year Publication Trends in Foot and Ankle Literature: Improved Methodological Quality and Internationality With Time

**DOI:** 10.5435/JAAOSGlobal-D-20-00154

**Published:** 2021-02-10

**Authors:** Derek M. Klavas, Jonathan Liu, Brendan M. Holderread, Jason S. Ahuero, Pedro E. Cosculluela, Kevin E. Varner, Joshua D. Harris

**Affiliations:** From the Houston Methodist Hospital (Dr. Klavas, Dr. Ahuero, Dr. Cosculluela, Dr. Varner, and Dr. Harris) and the Texas A&M University College of Medicine (Mr. Liu and Mr. Holderread), Houston, TX.

## Abstract

**Background:**

Methodological quality and author internationality are increasing in orthopaedic surgery. The purpose of this study was to evaluate the methodological quality and author geography trends from 1994 to 2019 in high-quality foot and ankle journals.

**Methods:**

Analyses of 1,242 foot and ankle publications in *Foot and Ankle International*, *American Journal of Bone and Joint Surgery*, and *American Journal of Sports Medicine* were done for 1994, 1999, 2004, 2009, 2014, and 2019. Articles were classified according to study type, level of evidence (LOE), and author's country of publication.

**Results:**

The most common clinical study was therapeutic (65.4). Significant increases were noted in the proportion of therapeutic (*P* < 0.01) and prognostic (*P* < 0.01) articles. The average LOE increased from 3.96 ± 1.01 to 3.19 ± 0.97 (*P* < 0.01). The proportion of Level I (*P* = 0.29) and level IV articles (*P* = 0.21) remained constant, level II (*P* < 0.01) and level III (*P* < 0.01) articles increased, and level V (*P* < 0.01) articles decreased. United States authorship decreased from 78.1% in 1994 to 44.8% in 2009, then remained constant through 2019 (*P* < 0.01).

**Conclusion:**

This study demonstrated an improvement in LOE of foot and ankle publications across a 25-year period in three high-quality orthopaedic journals. Increasing internationality was also observed.

The specialty of foot and ankle surgery is rapidly evolving as orthopaedic surgeons, researchers, and medical technology continue to improve subjective patient-reported and objective clinician-measured outcomes.^1^ Foot and ankle surgeons must adapt to changes in scientific innovation as they arise. One way is to make data-driven decisions using evidence-based medicine, a process of systematically evaluating and appraising clinical research findings to guide optimal clinical care to patients.^2^ As the volume of journals and publications dramatically increase, an increasing need exists for high-quality, easily accessible, evidence-based studies for busy clinicians.

Several dozen methodological quality and risk of bias scores used by multiple organizations exist, including EQUATOR and the Cochrane Collaboration, that grade the quality of and characterize the different types of medical publications. One of the most common quality assessments of an investigation is level of evidence (LOE). Because the advent of routine LOE assignment, multiple orthopaedic journals have reported an increase in the proportion of higher evidence articles.^3,4,5,6^ A study of orthopaedic literature in 2005 reported that foot and ankle studies averaged the lowest levels of evidence of the orthopaedic specialties evaluated.^7^ However, in a more recent investigation, Zaidi et al.^8^ showed that foot and ankle publications from 2000 to 2010 noted increasing LOE. No more recent studies exist on foot and ankle investigations within the past decade. Similarly, with the growth of multicenter and international collaboration in orthopaedic surgery, it is of interest to investigate the publication geography of orthopaedic specialties to improve publication international diversity. Unfortunately, a limited number of published studies exists, analyzing the internationality of foot and ankle literature.^8^

The purpose of the current study was to evaluate the methodological quality and author geography trends between 1994 and 2019 in multiple high-quality general and specialty-specific foot and ankle journals.

The study hypotheses are that (1) the proportion of level I and II evidence studies has increased from 1994 to 2019 and (2) the geographic internationality of authorship has increased from 1994 to 2019.

## Methods

Analyses of foot and ankle publications in three orthopaedic journals were done at six time points by examining 1-year time spans every 5 years (January 1, 1994, to December 31, 1994; January 1, 1999, to December 31, 1999; January 1, 2004, to December 31, 2004; January 1, 2009, to December 31; January 1, 2014, to December 31, 2014; and January 1, 2019, to December 31, 2019). All clinical and preclinical (cadaver, biomechanical, laboratory, and animal) techniques, systematic reviews, and meta-analyses were included. Letters to the editor, responses to letters to the editor, editorials, special topics, correspondence, and news announcements were excluded. Articles that were electronically published ahead of print during one of the above time periods were not included unless their corresponding print article was also released during the same year.

Three orthopaedic journals (two general orthopaedic journals and one subspecialty journal) that routinely publish foot and ankle literature were chosen based on their high impact factors (IF) as reported by the 2018 InCites Journal Citation Reports (Clarivate Analytics)^9^; Foot and Ankle International (FAI) (IF 2.341, 5-year IF 2.506), Journal of Bone and Joint Surgery–American Volume (JBJS-A) (IF 4.716, 5-year IF 6.101), and American Journal of Sports Medicine (AJSM) (IF 6.093, 5-year IF 7.006). A manual review of each issue's table of context for the selected calendar years was done. Each article included in the study was available in full text English language print form and electronically via the journal's website.

All included articles were independently reviewed by three authors (D.M.K., J.L., and B.M.H.). The articles were first designated as either clinical or preclinical. First author and last author's countries were recorded for all articles. In the event that more than one country was listed anywhere in the article's authorship line, this was recorded as “multiple countries” to capture true authorship diversity. Clinical articles were then evaluated further for their research design, including prospective versus retrospective methodology, use of a control group, randomization, number of participants, average follow-up, or use of a large national database/registry. This information was subsequently used to determine study type and LOE according to the JBJS-A grading system.^10^ Preclinical articles were not classified any further because their design does not allow for LOE rating. Study type was classified as per the Center for Evidence-Based Medicine from the University of Oxford: therapeutic (pertaining to the results of a treatment), prognostic (pertaining to the outcome of a disease process), diagnostic (pertaining to a diagnostic test), or economic (pertaining to cost or economic decision model) outcomes.^11^ LOE was assigned on a scale of I (highest) to V (lowest). In the infrequent event that the reviewer's assigned LOE grade differed from the LOE grade reported directly by the journal, the study was reanalyzed as group including the senior author (J.D.H.) and a revised LOE grade was given.

Descriptive statistics were calculated for all data and presented as means with SDs. Student *t* tests and single factor analysis of variance were used to compare the means of continuous data. Chi squared analysis and Fisher exact tests were used to compare categorical data—specifically the proportions of study type and LOE by both year of publication and journal type. Statistical significance was defined as *P*-value < 0.05. All analyses were done using SPSS (Statistical Packages for the Social Sciences) software (IBM).

## Results

### Article and Study Type

**Figure 1:**
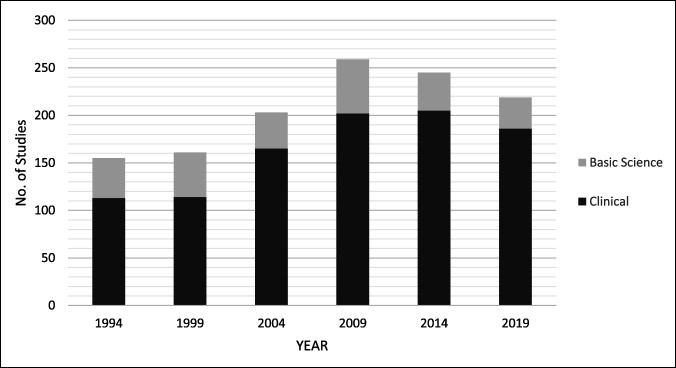
Graphical representation demonstrating the number of preclinical articles versus number of clinical articles by year of publication for all included studies (n = 1,242).

A total of 1,242 articles were included for analysis across the six time points which can be seen in Table 1 and Figure 1. The total number of articles increased steadily between each time point, with the largest increase occurring between 2004 (203 articles) and 2009 (259 articles). An overall increase was observed in the proportion of published clinical articles to preclinical articles during the study period (*P* < 0.01), with the largest discrepancy occurring in 2019—15% preclinical studies versus 85% clinical studies (Figure 1).

**Table 1:** Distribution of Preclinical (Cadaver, Biomechanical, Laboratory, and Animal) Articles Versus Clinical Articles by Year for all Included Studies (n = 1,242)

Study Type	All included studies (n = 1,242)						
	1994	1999	2004	2009	2014	2019	*P* Value
Preclinical	42 (27.1)	47 (29.2)	38 (18.7)	57 (22.0)	40 (16.3)	33 (15.1)	**<0.01**
Clinical	113 (72.9)	114 (70.8)	165 (81.3)	202 (78.0)	205 (83.7)	186 (84.9)	

p-value = 0.01.

**Figure 2:**
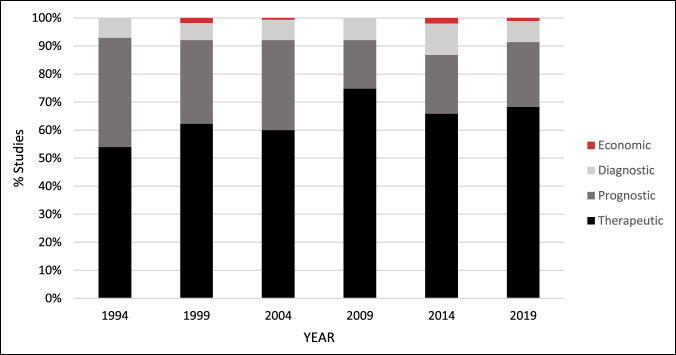
Graphical representation demonstrating the study type by year of publication for all clinical studies analyzed (n = 985).

A total of 985 clinical studies were analyzed for study type which can be seen in Table 2 and Figure 2. The most common was therapeutic (65.4%), followed by prognostic (25.6%), diagnostic (8.1%), then economic (0.9%) types. Statistically significant increases were noted in the number of therapeutic (*P* < 0.01) and prognostic (*P* < 0.01) articles across the six time points (Table 2). No difference was noted in the proportion of prognostic (*P* = 0.59) nor economic (*P* = 0.23) studies across the six time points (Table 2). Twelve clinical studies (1.22%) used a large national database for their data collection: one from 1994, two from 2009, four from 2014, and five from 2019.

**Table 2:** Distribution of all Clinical Articles (n = 985) by Study Type and Level of Evidence, by Year of Publication

Study Type	Clinical Articles (n = 985)^a^						
	1994	1999	2004	2009	2014	2019	*P* Value
Clinical							
Therapeutic	61 (54.0)	71 (62.3)	99 (60.0)	151 (74.8)	135 (65.9)	127 (68.3)	**<0.01**^b^
Prognostic	44 (38.9)	34 (29.8)	53 (32.1)	35 (17.3)	43 (21.0)	43 (21.0)	**<0.01**^b^
Diagnostic	8 (7.1)	7 (6.1)	12 (7.3)	16 (7.9)	23 (11.2)	14 (7.5)	0.59^b^
Economic	0 (0.0)	2 (1.8)	1 (0.6)	0 (0.0)	4 (1.9)	2 (1.1)	0.23^c^
LOE							
I	4 (3.5)	2 (1.8)	10 (6.1)	7 (3.5)	13 (6.3)	12 (6.5)	0.29^c^
II	6 (5.3)	5 (4.4)	13 (7.9)	12 (5.9)	27 (13.2)	29 (15.6)	**<0.01**^b^
III	18 (15.9)	14 (12.3)	38 (23.0)	41 (20.3)	60 (29.3)	66 (35.5)	**<0.01**^b^
IV	47 (41.6)	55 (48.2)	62 (37.6)	86 (42.6)	71 (34.6)	70 (37.6)	0.21^b^
V	38 (33.6)	38 (33.3)	42 (25.4)	56 (27.7)	34 (16.6)	9 (4.8)	**<0.01**^b^

LOE = level of evidence. All bolded p-values = 0.01.

a Values displayed as n (%).

b Chi squared analysis.

c Fisher exact test.

### Level of Evidence

**Figure 3:**
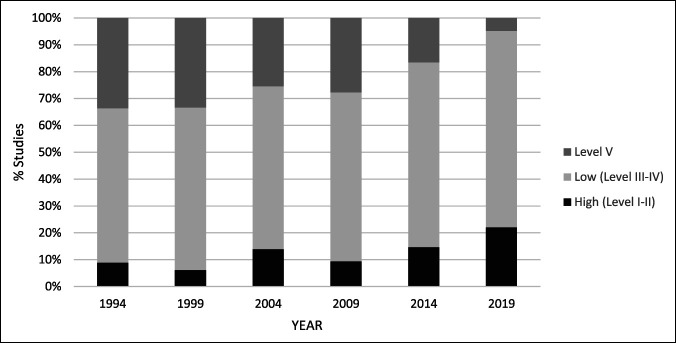
Graphical representation demonstrating the level of evidence (LOE) by year of publication for all clinical studies analyzed (n = 985). High LOE are defined by levels I and II and low LOE are defined by levels III and IV. Level V studies displayed separately.

Overall, the average LOE improved from 3.96 ± 1.01 in 1994 to 3.19 ± 0.97 in 2019 (*P* < 0.01). LOE breakdown by year can be seen in Table 2 and Figure 3. Most articles across the six time points were level III (24.1%), level IV (39.7%), or level V (22.0%) evidence. Level I (4.9%) and level II (9.3%) evidence composed the minority of articles. The proportion of high level (I and II) evidence increased significantly from 8.8% in 1994 to 22.0% in 2019 (*P* < 0.001), whereas the proportion of low level (III and IV) evidence varied between 57.5% in 1994 to 62.8% in 2009, then increased to 73.1% by 2019 (*P* = 0.032).

**Figure 4:**
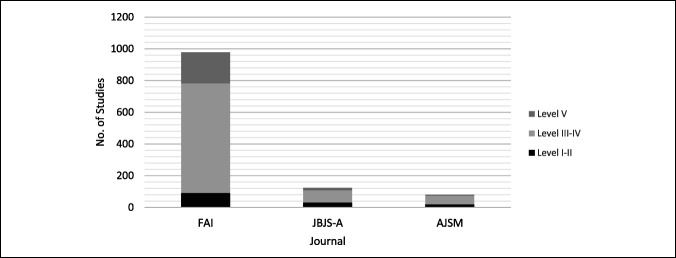
Graphical representation demonstrating the level of evidence (LOE) for all clinical studies analyzed (n = 985) subdivided by journal of publication. High LOE are defined by levels I and II and low LOE are defined by levels III and IV. Level V studies displayed separately. AJSM = American Journal of Sports Medicine (n = 80), FAI = Foot and Ankle International (n = 782), and JBJS-A = Journal of Bone and Joint Surgery–American Volume (n = 123).

LOE breakdown by journal can be seen in Table 3 and Figure 4. FAI contributed 79.4% of all clinical articles. The proportion of therapeutic (*P* < 0.01) and prognostic (*P* < 0.01) studies significantly increased across the six study time points. JBJS-A and AJSM contributed 12.5% and 8.1% of all clinical articles, respectively. The proportion of therapeutic studies in JBJS-A significantly increased across the study period; however, the proportion of high-level studies did not differ (*P* = 0.08). AJSM did not demonstrate a notable change over time regarding the proportion of study type or LOE. Large database studies composed 4.3% of all level II articles, 2.5% of all level III articles, and 0.5% of all level IV articles.

**Table 3:** Distribution of all Clinical Articles (n = 985) by Study Type and Level of Evidence by the Journal of Publication

Study Type	FAI (n = 782)^a^						
	1994	1999	2004	2009	2014	2019	*P* Value
Clinical							
Therapeutic	52 (57.1)	59 (60.2)	71 (58.7)	122 (76.7)	101 (63.9)	103 (66.4)	**<0.01**^b^
Prognostic	32 (35.2)	30 (30.6)	41 (33.9)	25 (15.7)	36 (22.8)	37 (23.9)	**<0.01**^b^
Diagnostic	7 (7.7)	7 (7.1)	9 (7.4)	12 (7.6)	18 (11.4)	13 (8.4)	0.79^b^
Economic	0 (0.0)	2 (2.1)	0 (0.0)	0 (0.0)	3 (1.9)	2 (1.3)	0.18^c^
LOE							
High (I-II)	4 (4.4)	5 (5.1)	17 (14.0)	12 (7.6)	23 (14.6)	29 (18.7)	**<0.01**^c^
Low (III-V)	87 (95.6)	93 (94.9)	104 (86.0)	147 (92.4)	135 (85.4)	126 (81.3)	

	JBJS-A (n = 123)^a,c^						
	1994	1999	2004	2009	2014	2019	*P* Value
Clinical							
Therapeutic	5 (41.7)	10 (83.3)	23 (82.2)	20 (87.1)	24 (75.0)	15 (93.8)	**0.03**
Prognostic	7 (58.3)	2 (16.7)	2 (7.1)	1 (4.3)	3 (9.4)	0 (0.0)	**<0.01**
Diagnostic	0 (0.0)	0 (0.0)	2 (7.1)	2 (8.6)	5 (15.6)	1 (6.2)	0.65
Economic	0 (0.0)	0 (0.0)	1 (3.6)	0 (0.0)	0 (0.0)	0 (0.0)	0.74
LOE							
High (I-II)	2 (16.7)	1 (8.3)	4 (14.3)	4 (17.4)	13 (40.6)	6 (37.5)	0.08
Low (III-V)	10 (83.3)	11 (91.7)	24 (85.7)	19 (82.6)	19 (59.4)	10 (62.5)	

	AJSM (n = 80)^a,c^						
	1994	1999	2004	2009	2014	2019	*P* Value
Clinical							
Therapeutic	4 (40.0)	2 (50.0)	5 (31.3)	9 (45.0)	10 (66.7)	9 (60.0)	0.41
Prognostic	5 (50.0)	2 (50.0)	10 (62.5)	9 (45.0)	4 (26.7)	6 (40.0)	0.51
Diagnostic	1 (10.0)	0 (0.0)	1 (6.2)	2 (0.1)	0 (0.0)	0 (0.0)	0.63
Economic	0 (0.0)	0 (0.0)	0 (0.0)	0 (0.0)	1 (6.6)	0 (0.0)	0.55
LOE							
High (I-II)	4 (40.0)	1 (25.0)	2 (12.5)	3 (15.0)	4 (26.7)	6 (40.0)	0.34
Low (III-V)	6 (60.0)	3 (75.0)	14 (87.5)	17 (85.0)	11 (73.3)	9 (60.0)	

AJSM = American Journal of Sports Medicine (n = 80), FAI = Foot and Ankle International (n = 782), JBJS-A = Journal of Bone and Joint Surgery–American Volume (n = 123), LOE = level of evidence

a Values displayed as n (%).

b Chi squared analysis.

c Fisher exact test.

High LOE defined by levels I and II and low LOE defined by levels III through V. All bolded p-values = 0.01.

### Authorship

**Figure 5:**
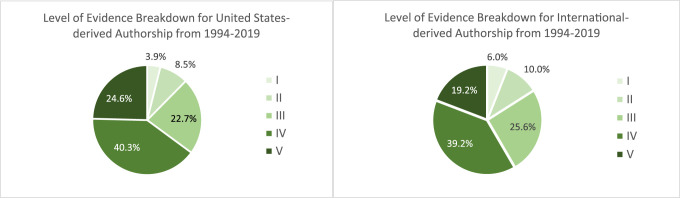
Graphical representation demonstrating the level of evidence distribution for all clinical studies analyzed (n = 985), subdivided by United States–derived authorship (n = 517) and International authorship (n = 469). International authorship was defined as having at least one author who's country of origin was outside the United States.

**Figure 6:**
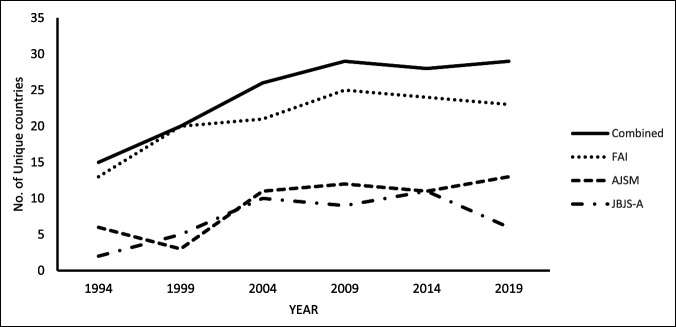
Graphical representation demonstrating the number of unique countries represented by each journal for all included studies (preclinical and clinical studies, n = 1,242). AJSM = American Journal of Sports Medicine (n = 111), FAI = Foot and Ankle International (n = 978), and JBJS-A = Journal of Bone and Joint Surgery–American Volume (n = 153)

The average number of authors per study was 4.3 ± 2.0. For clinical studies only, the average number of authors per study increased consistently from 3.1 ± 1.3 in 1994 to 3.8 ± 1.9 in 2009 to 5.6 ± 2.1 in 2019 (*P* < 0.01) (Table 4). Authorship breakdown by the country of origin and year can be seen in Table 5. For all included studies, United States–derived authorship decreased from 78.1% in 1994 to 44.8% in 2009, then remained between 45.3% and 50.9% through 2019 (*P* < 0.01) (Table 5). For only clinical studies, United States–derived authorship decreased from 77.0% in 1994 to 43.6% in 2009, then increased to 46.8% and 51.1% in 2014 and 2019, respectively (*P* < 0.01) (Table 4). United States–derived authorship showed an average LOE of 3.7 ± 1.0, compared with international authorship that demonstrated an average LOE of 3.5 ± 1.1 (*P* < 0.01). Breakdown of LOE grade between United States authorship and international authorship can be seen in Figure 5. High-grade evidence similarly comprised 13% of US-written articles and 16% of internationally written articles. Articles with authors representing two or more countries increased dramatically throughout the study period, with a peak in 2014 that yielded 36 articles with authors from two or more countries. The five largest international contributors overall were the United Kingdom, South Korea, Japan, Germany, and Canada. Articles originated from a total of 42 unique countries with the number of countries represented per year increasing from 16 in 1994 to 27 in 2004, peaking at 29 in 2014 and 2019. FAI had the largest international representation of the three journals, ranging from 11 countries in 1994 to 24 countries in 2014 (Figure 6).

**Table 4:** Breakdown of United States Authorship Versus International Authorship for All Studies (Preclinical, n = 257; Clinical, n = 985)

Authorship	Preclinical	Clinical					
	1999-2019	1994	1999	2004	2009	2014	2019
Ave. authors (n ± SD)	4.63 ± 1.8	3.12 ± 1.3	3.61 ± 2.0	3.52 ± 1.6	3.82 ± 1.9	4.81 ± 1.9	5.62 ± 2.1
Only United States authors (n)	175	87	66	90	88	96	95
Any non-United States authors (n)	82	26	48	75	114	109	91
Multiple countries^a^ (n)	26	1	5	5	10	31	22
% Only United States authors	68.1	77.0	57.9	54.5	43.6	46.8	51.1

a Indicates articles that listed authors from ≥ 2 countries, including United States.

Percentage of only US authors calculated by the number of articles with only US authorship divided by the number of articles with at least one non-US author.

**Table 5:** Breakdown of Authorship by Individual Country for All Included Studies (n = 1,242)

Country	1994	1999	2004	2009	2014	2019
Argentina	0 (0.00)	0 (0.00)	2 (0.99)	0 (0.00)	0 (0.00)	1 (0.46)
Australia	0 (0.00)	8 (4.97)	6 (2.96)	9 (3.47)	3 (1.22)	2 (0.91)
Austria	1 (0.65)	2 (1.24)	3 (1.48)	1 (0.39)	1 (0.41)	4 (1.83)
Belgium	1 (0.65)	3 (1.86)	1 (0.49)	1 (0.39)	2 (0.82)	1 (0.46)
Brazil	0 (0.00)	1 (0.62)	0 (0.00)	0 (0.00)	2 (0.82)	1 (0.46)
Canada	5 (3.23)	3 (1.86)	3 (1.48)	6 (2.32)	7 (2.86)	10 (4.57)
Chile	0 (0.00)	0 (0.00)	0 (0.00)	0 (0.00)	1 (0.41)	2 (0.91)
China	0 (0.00)	0 (0.00)	1 (0.49)	5 (1.93)	9 (3.67)	4 (1.83)
Costa Rica	0 (0.00)	0 (0.00)	0 (0.00)	1 (0.39)	0 (0.00)	0 (0.00)
Croatia	0 (0.00)	0 (0.00)	0 (0.00)	1 (0.39)	0 (0.00)	0 (0.00)
Denmark	2 (1.29)	3 (1.86)	1 (0.49)	0 (0.00)	2 (0.82)	1 (0.46)
Egypt	0 (0.00)	0 (0.00)	0 (0.00)	1 (0.39)	0 (0.00)	3 (1.37)
Finland	1 (0.65)	0 (0.00)	1 (0.49)	1 (0.39)	3 (1.22)	1 (0.46)
France	0 (0.00)	3 (1.86)	0 (0.00)	5 (1.93)	1 (0.41)	2 (0.91)
Germany	2 (1.29)	4 (2.48)	9 (4.43)	12 (4.63)	5 (2.04)	5 (2.28)
Greece	2 (1.29)	0 (0.00)	1 (0.49)	5 (1.93)	0 (0.00)	1 (0.46)
Hong Kong	0 (0.00)	0 (0.00)	0 (0.00)	0 (0.00)	1 (0.41)	0 (0.00)
Hungary	0 (0.00)	0 (0.00)	1 (0.49)	0 (0.00)	0 (0.00)	0 (0.00)
India	0 (0.00)	1 (0.62)	0 (0.00)	0 (0.00)	1 (0.41)	0 (0.00)
Iran	0 (0.00)	0 (0.00)	0 (0.00)	0 (0.00)	0 (0.00)	1 (0.46)
Ireland	2 (1.29)	0 (0.00)	2 (0.99)	0 (0.00)	0 (0.00)	0 (0.00)
Israel	0 (0.00)	4 (2.48)	1 (0.49)	3 (1.16)	4 (1.63)	0 (0.00)
Italy	3 (1.94)	2 (1.24)	6 (2.96)	6 (2.32)	4 (1.63)	1 (0.46)
Jamaica	0 (0.00)	0 (0.00)	1 (0.49)	0 (0.00)	0 (0.00)	0 (0.00)
Japan	3 (1.94)	8 (4.97)	13 (6.40)	9 (3.47)	6 (2.45)	7 (3.20)
Multiple countries^b^	2 (1.29)	10 (6.21)	5 (2.46)	12 (4.63)	36 (14.69)	25 (11.42)
Netherlands	0 (0.00)	1 (0.62)	5 (2.46)	2 (0.77)	5 (2.04)	6 (2.74)
New Zealand	0 (0.00)	0 (0.00)	0 (0.00)	1 (0.39)	1 (0.41)	0 (0.00)
Norway	1 (0.65)	0 (0.00)	1 (0.49)	0 (0.00)	1 (0.41)	0 (0.00)
Poland	0 (0.00)	0 (0.00)	0 (0.00)	0 (0.00)	0 (0.00)	1 (0.46)
Portugal	0 (0.00)	0 (0.00)	0 (0.00)	2 (0.77)	0 (0.00)	0 (0.00)
Singapore	0 (0.00)	0 (0.00)	1 (0.49)	1 (0.39)	1 (0.41)	0 (0.00)
Slovenia	0 (0.00)	1 (0.62)	0 (0.00)	0 (0.00)	0 (0.00)	0 (0.00)
South Africa	1 (0.65)	0 (0.00)	0 (0.00)	1 (0.39)	0 (0.00)	0 (0.00)
South Korea	0 (0.00)	0 (0.00)	1 (0.49)	16 (6.18)	16 (6.53)	15 (6.85)
Spain	1 (0.65)	2 (1.24)	0 (0.00)	2 (0.77)	1 (0.41)	2 (0.91)
Sweden	4 (2.58)	0 (0.00)	3 (1.48)	1 (0.39)	1 (0.41)	0 (0.00)
Switzerland	0 (0.00)	3 (1.86)	10 (4.93)	11 (4.25)	4 (1.63)	4 (1.83)
Taiwan	0 (0.00)	2 (1.24)	1 (0.49)	2 (0.77)	0 (0.00)	1 (0.46)
Thailand	0 (0.00)	0 (0.00)	1 (0.49)	2 (0.77)	1 (0.41)	1 (0.46)
Turkey	0 (0.00)	1 (0.62)	5 (2.46)	1 (0.39)	2 (0.82)	2 (0.91)
United Kingdom	3 (1.94)	4 (2.48)	6 (2.96)	23 (8.88)	13 (5.31)	4 (1.83)
United States	121 (78.06)	95 (59.01)	112 (55.17)	116 (44.79)	111 (45.31)	111 (50.86)
Total countries	16	20	27	29	28	29

a Values displayed as n (%).

b Indicates articles that listed authors from ≥ 2 countries, including United States.

## Discussion

The primary outcome of this study was an improvement in LOE of foot and ankle publications across a 25-year eligibility period in three high quality orthopaedics journals, confirming the study's primary hypothesis. Increasing geographic diversity, or internationality, of author institution of publication was also observed, confirming the study's secondary hypothesis. The proportion of clinical studies (relative to preclinical) increased over the eligibility period. The mainstay of clinical research articles were therapeutic, followed by prognostic, and then diagnostic. Economic studies were poorly represented across all time points.

Animal, cadaver, and laboratory preclinical articles saw a statistically notable decrease in publication across the study period. In vitro studies play an important role in mimicking the environment that leads to a disease process, although simultaneously advancing research and development of potential therapies.^12^ Less representation of these types of publication is not a reflection of decreased importance, but perhaps a shift in the amount of time and resources readily available to busy orthopaedic surgeons to conduct laboratory research. Another possible explanation for the notable decrease of published in vitro studies is an increased effort by orthopaedic journals placed on the study of human subjects and subjective patient-reported clinical outcomes. A 2010 study of publication trends in the foot and ankle literature from seven orthopaedic and podiatric journals yielded 117 clinical research articles of 245 total (47.8%) journals.^13^ This number is notable lower than the percentage of clinical studies identified by the current study (range 70.8% to 84.9%), which is likely because of their exclusion of both level V articles and review articles.

The use of large databases or patient registries to study patient outcomes has increased tremendously in recent years. However, these studies are commonly associated with limitations including inaccurate data collection, inconsistent patient sampling methods, incomplete follow-up, and lack of orthopaedic-specific information.^14^ Despite these limitations, large database studies often receive higher LOE grades because of their reporting on patient-reported outcome measures with high statistical power. Between the years of 1995 and 2015, Bohl et al^14^ found only 13 foot and ankle publications from major orthopaedic journals that used nationwide administrative databases, which notably trailed the subspecialties of spine and joint arthroplasty surgeries. The current study found a similar paucity of large database usage in the foot and ankle literature with less than five percent of level II-IV studies using these methods.

The improvement of LOE observed in the current study is because of a higher percentage of level II and III articles and a lower percentage of level V articles, whereas the percentage of level I and IV articles remained constant (Table 2). The current study found that high level RCTs composed between 6.1% and 6.5% of all clinical articles since 2004, which is similar to the rate in orthopaedic trauma literature^15^ and orthopaedic sports medicine literature.^3,16,17^ Increased proportion of high LOE in recent years could be explained, in part, by a change in editorial policies at high IF journals. Both JBJS-A and FAI have made concerted efforts to limit the number of low level studies published. Established in 2011, JBJS case connector was created by JBJS-A to exclusively publish case report studies. In 2016, open access journals such as *JBJS Open Access* and *Foot and Ankle Orthopaedics* were created. Open access forums such as these allow for authors to garner academic visibility through shortened publication times and higher acceptance rates.^18^ Similarly, the field of orthopaedic sports medicine has added the international online-only *Open Access Journal of Sports Medicine* that began publishing in 2010.

Despite the trend toward improved LOE in the foot and ankle literature, the overwhelming majority of evidence is still made up of level III to V evidence (Figure 3). This is not inconsistent with most orthopaedic research. A number of explanations for the lack of high-level evidence exist. Nontraumatic orthopaedic procedures are elective in nature and frequently involve a shared-decision making process that assimilates a patient's preferences and a surgeon's recommendation. High-quality randomized trials in surgical interventions are incredibly challenging to do, with or without a natural history control or sham/placebo group. This is because of several reasons: patients are highly educated based on publicly available medical literature and have preconceived notions that preclude randomization and allocation.

Ultimately, lower level (III and IV) retrospective evidence continue to serve a purpose in orthopaedic literature. However, the limitations of these studies must be recognized, mitigated, and discussed in the interpretation of the clinical relevance. The reason that these investigations continue to predominate orthopedic literature (versus cardiovascular medicine, oncologic, and other medical specialties) is clearly multifactorial, but likely because of a “quantity over quality” culture in medical publishing. In addition, retrospective investigations require less time, resources (financial and nonfinancial), and are easy to complete under time constraints of a busy academic surgeon.^3^ Another commonly cited reason is that retrospective studies can be published expeditiously by residents and medical students who wish to expand their curriculum vitae for career purposes.^19^ The current study found that level IV articles remained constant over the 25-year study period. This is similar to a previous foot and ankle publication trend study published by Zaidi et al.^8^ who found that level IV articles remained constant between 46 and 50% between 2000 and 2010.

Overall improvement in LOE of clinical articles across the study period is paralleled by increasing journal IF of the three studied journals. Journal IFs are a general marker of the overall impact of the journal on scientific research from a given specialty. Currently, FAI holds an IF of 2.341 and has increased over 500% from 0.452 because IFs were first recorded in 1997. The current IF for JBJS-A is 4.716 and has more than doubled from 2.190, whereas AJSM is the highest rated orthopaedic journal with a current IF of 6.093 up 380% from 1.605.^9^ For reference, the mean IF for general orthopaedic journals (such as AJSM and JBJS-A) and specialized orthopaedic journals (such as FAI) increased from 1.4 in 2010 to 1.9 in 2016.^20^ In the previous decade, the number of orthopaedic specialty journals increased, from 24 to 41, more than any other specialty. However, the mean IF rank of orthopaedic specialty journals decreased from sixth to seventh of the nine surgical subspecialties.^21^

As seen in Table 4, the number of authors per clinical research article increased from 3.1 to 5.6 over the 25-year study period. This pattern has been observed elsewhere in the orthopaedic literature because the mean number of authors in orthopaedic articles from JBJS and CORR increased from 1.6 authors per article in 1958 to 4.1 authors per article in 2008.^22^ More authors per article may represent multicenter investigations and collaborative efforts among leaders in the field. However, the dispute can also be made that increased authorship is reflective of unfitting “quid pro quo” authorship to boost the reputation of one's associates.

This increase in the number of authors per article is paralleled by a non-US authorship increase by 28% over the study period (Table 4). Table 4 depicts the prevalence of first and last authors from each country listed, by year. Lead author and senior author geographic affiliations were analyzed because they are the positions most responsible for the bulk of research design, execution, and study preparation. In addition, low likelihood exists for a study to be done in a country outside of either affiliation listed by the first and last author on a study. The three journals included in this study are known to have a large international readership that is reflected within the geographic variation found in this study. For example, 2014 and 2019, respectively, saw 14.7% and 11.4% of the publications list authors from multiple countries. Furthermore, as shown in Figure 5, international authors produced a similar proportion of high-level studies as their US counterparts and a smaller proportion of level V evidence. Of particular mention is the increased contribution from South Korea, who has increased its contribution to high impact foot and ankle literature by 6.5% since 2004. Only the United Kingdom demonstrated a similar increase in contribution, which was 6.9% between the years of 1994 and 2009 (Table 5).

The current study has several strengths pertaining to the manner in which our analysis was done. The data encompass publication trends over 25 years, as opposed to a single snapshot in time. In addition, the journals included in this study are two of the highest impact general orthopaedic journals and the single highest impact foot and ankle-specific journal. Finally, multiple independent reviewers used standardized criteria to identify study type and LOE ratings which have been shown in previous studies to have reliable interobserver agreement.^8,23^

A number of limitations exist in the current study. Only three orthopaedic journals were surveyed—one specific to the foot and ankle literature and two general orthopaedic journals. There may be differing trends in other journals that were not represented, specifically foot and ankle journals with lower IFs. Next, by studying articles from one calendar year over 5-year intervals, it is possible that our review missed certain trends that occurred during other time points. However, evaluating articles from every year would be excessively time consuming and would not likely yield additional helpful information. Finally, we did not evaluate the methodological quality (eg, Coleman, CLEAR-NPT, Jadad, MINORS, CONSORT, etc) or risk of bias (eg, RoB2 and ROBINS-I) of the included articles which can provide valuable information regarding study validity and trends of quality within a given LOE rank.

## Conclusion

The orthopaedic subspecialty of foot and ankle surgery is rapidly growing with increasingly more fellowship-trained surgeons doing a growing number of procedures each year. To match this growth, the foot and ankle orthopaedic literature should continue to expand to allow its surgeons to make data-driven decisions when providing clinical care. This study demonstrated an improvement in LOE of over 1,200 foot and ankle publications across a 25-year eligibility period in three high-quality orthopaedic journals. The proportion of clinical studies (relative to preclinical) increased, therapeutic studies were the most common clinical study type, followed by prognostic then diagnostic studies, whereas economic studies were poorly represented. Large database/registry studies represented a minority (<2%) of clinical studies. Increasing internationality of publication was also observed, highlighting the global presence of FAI, JBJS-A, and AJSM. Similar LOE distribution was observed between US and International authorship.
